# Magnetic graphene oxide increases the biocompatibility and nuclear factor erythroid 2-related factor 2 antioxidant of human cumulus cells: A lab-trial study

**DOI:** 10.18502/ijrm.v22i9.17475

**Published:** 2024-11-14

**Authors:** Fahimeh Kabiri, Tahereh Foroutan, Maryam Pashaiasl

**Affiliations:** ^1^Department of Animal Biology, Faculty of Biological Sciences, Kharazmi University, Tehran, Iran.; ^2^Department of Reproductive Biology, Faculty of Advanced Medical Sciences, Tabriz University of Medical Sciences, Tabriz, Iran.; ^3^Women's Reproductive Health Research Center, Tabriz University of Medical Sciences, Tabriz, Iran.

**Keywords:** Magnetic graphene oxide, Cumulus cells, Fertility, Nrf-2, Western blot.

## Abstract

**Background:**

Although assisted reproductive technology has been improved, the success rate is only 30%. Since the interaction between oocytes and cumulus cells (CCs) is necessary for the formation of a fertile oocyte, increasing the survival rate of CCs can improve the function of oocytes in infertile women.

**Objective:**

This study aimed to investigate the effects of magnetic graphene oxide (MGO) nanocomposite on the biocompatibility and antioxidant activity of human CCs.

**Materials and Methods:**

In this lab-trial study, from July 2021–2023 human CCs were collected from 37 women aged 20–37 yr and cultured in a medium containing Dulbecco's Modified Eagle's/F12, fetal bovine serum (10%), and penicillin-streptomycin (1%). Then CCs were treated with increasing concentrations of nano-MGO for 24, 48, and 72 hr (3[4, 5-dimethylthiazole-2-yl]-2, 5-diphenyltetrazolium bromide) assay and flow cytometry technique were used to compare the survival rate and apoptosis of CCs before and after treatment. Western blot test was used for expressing nuclear factor erythroid 2-related factor 2 (Nrf2) antioxidant in 2 groups.

**Results:**

The results of the present study showed that treatment with MGO increased the viability of CCs at a concentration of 50 µg/ml after 48 hr (p > 0.01). At higher doses (100 µg/ml) MGO decreased the survival rate of CCs (p > 0.05). Also, treatment with MGO at a concentration of 50 µg/ml increased the expression level of antioxidant protein Nrf2 in human CCs.

**Conclusion:**

Our results highlight the use of MGO in a new strategy that improves CCs viability and secretion of antioxidant protein Nrf2, thereby potentially increasing in vitro fertilization outcomes.

## 1. Introduction

Recently, several studies have been conducted to find non-invasive tools for indirect evaluation of oocyte quality, focusing on cumulus cells (CCs) as a mirror of oocyte characteristics (1, 2). For the formation of a suitable and fertile oocyte, the interaction between the oocyte and CCs is necessary to lead to a successful pregnancy (1). CCs affect oocyte maturation by releasing secretory signals followed by the resumption of meiosis and cytoskeleton changes (2). Oocyte competence is defined as the inherent ability of the oocyte to undergo the maturation of female gamete, fertilization, and embryogenesis (3, 4). As damage to the CCs reduces the rate of fertilization and blastocyst formation, increasing its biological potential can enhance oocyte capabilities (5).

Today, magnetic graphene oxide (MGO) has been introduced as one of the attractive biomaterials with excellent biocompatibility and nontoxic properties for biomedical and regenerative medicine (6). There has been no research yet about the applications of MGO in the field of infertility couples. The main objective of this research was to study the effects of MGO utilization for possible increases in biocompatibility and antioxidant properties of CCs in infertile couples. Moreover, MGO nanoparticles are able to absorb proteins such as bone morphogenetic protein 2, to differentiate osteoblasts from osteocytes (7). On the other hand, the effects of accelerating stem cell differentiation of MGO are more than graphene oxide (GO) (8). MGO is a material composed of GO that has the characteristics of GO and magnetic material. It has been showed that the properties of MGO are correlated with dose and reaction period (9). On the other hand, some GO derivatives have antioxidant activity (10). Nuclear factor erythroid 2-related factor 2 (Nrf2) is an important protein for increasing the level of many antioxidants within the body.

Since the relationship between apoptosis and the survival rate of human CCs has been considered as markers of oocyte quality for selecting the most appropriate oocytes in in vitro fertilization (IVF) the use of biomaterials that improve the quality of CCs can be a way to improve the quality of oocytes (11). Previous study showed that intraperitoneal injection of MGO can increase in vivo maturation of oocytes (12). In this study for the first time, we investigated the effects of MGO on viability and antioxidant activity of human CCs by 3[4, 5-dimethylthiazole-2-yl]-2, 5-diphenyltetrazolium bromide (MTT) assay method. Also, the effects of MGO on apoptosis and antioxidant activity of human CCs were studied.

## 2. Materials and Methods 

### Study design

This lab-based trial study involved the follicular fluid of 37 women with male factor infertility who were referred to Al-Zahra hospital Tabriz, Iran between July 2021–2023. Women aged between 20 and 37 yr with male factor infertility were included in this study. An attempt was made to use CCs from women in which > 80% of their oocytes were in the metaphase II stage. Women with specific diseases such as diabetes and heart disease were excluded from the study.

### Isolation and culture of human CCs

#### Follicular aspiration

Follicular fluid from women referred for puncture to do intracytoplasmic sperm injection was obtained by a gynecologist. Briefly, transvaginal oocyte retrieval was carried out under ultrasound guidance with intravenous sedation, typically scheduled 36 hr post-administration of human chorionic gonadotropin. The follicular fluid was evaluated by a stereo microscope, CCs surrounding the oocyte-cumulus complexes of each patient were mechanically separated and transferred to a sterile microtube containing culture medium by a reproductive biologist.

### Primary culture of CCs

CCs extracted from patients were washed twice in a culture medium and then centrifuged at 1600 rpm for 10 min at room temperature. Viability assessment and cell counting were performed using Trypan Blue. The CCs were cultured in Dulbecco's Modified Eagle's/F12 medium (DMEM/F12) (Bio-Idea Co., Iran) supplemented with fetal bovine serum (10%) (Gene X Co., Iran), and 1% penicillin/streptomycin (Bio-Idea Co., Iran) at 37 C with 5% CO ${\;}_{2}$ and 95% humidity. Whenever the CCs proliferated to passage 4, they would be characterized: MTT assay, Flow cytometry, and Western blot were performed.

### Synthesis of the MGO nanoparticles

In our previous study, we developed a simple method for the synthesis of MGO nanoparticle with a mean diameter of 200–400 nm and considered its application to improve the viability of isolated human CCs cultured in medium (13).

### MTT assay

The MTT assay was used to evaluate the cytotoxicity of MGO on CCs described by Chang et al., (14). Briefly, 100 µl of cell suspension (10,000 cells in complete DMEM/F12 containing 10% fetal bovine serum) was added to each well of a 96-well flat-bottomed plate. After 24 hr, required for cell adhesion, serial dilution of MGO (1.56, 3.125, 6.25, 12.5, 25, 50, 100, 200, and 400 µg/ml) were added on wells. Then 3 wells were considered as control and received 100 µl of cells, with culture medium. CCs were incubated for 24, 48, and 72 hr at 37 C and 5% CO ${\;}_{2}$ (15). Then, 20 µl of MTT (Cat. No. M5655, Sigma., USA) solution (5 mg/ml in PBS) was added to each well and were incubated for 4 hr. During this incubation period, the succinate dehydrogenase enzyme present in the mitochondria of healthy and viable cells reduces MTT to insoluble purple formazan crystals. Finally, the purple formazan crystals formed in the cytoplasm of the cells were dissolved by adding 100 µl of pure DMSO solution to the wells and placing the plates in a shaker incubator for 10–15 min. The optical density of CCs was measured by an ELISA reader (Bio Tek Instruments, Winooski, VT) at 570 nm wavelength. All steps were repeated at least 3 times.

### Cell apoptosis detection by anexin V-FITC/ propidium iodide (PI) staining

CCs (3 × 10^5^ cell/well) were seeded in 6-well plates for 24 hr and then different concentrations of MGO (3.12, 12.5, 50, and 100 µg/ml) were added to CCs. The control group did not receive any treatment. After 48 hr, the ApoFlowExⓇfluorescein isothiocyanate (FITC) kit from Exbio (Vestec, Czech Republic) was used for detection of apoptosis. According to manufacturer's protocol, the treated and untreated groups were trypsinized and resuspended in cold PBS. Then annexin-binding buffer was added to the cell suspension and stained with 5 µL annexin and 5 µL PI (ApoFlowExⓇFITC Kit). Then stained cells were incubated for 15 min at room temperature in darkness. Finally, cells were analyzed by flow cytometry.

### Western blotting

The total protein of the CCs was quantified using Bradford assay after 48 hr treatment with 50 µg/ml MGO. To separate the protein fraction sodium dodecyl-sulfate polyacrylamide gel electrophoresis (2.5%) was used. This fraction transferred to polyvinylidene fluoride membranes (Roche, UK) and blocked with bovine serum albumin in Tris-buffered saline at room temperature for 2 hr. After incubation of the membranes with primary antibodies (Santa Cruz Biotechnology, USA) against, Nrf2 (sc-365949), and β -actin (sc-517582) for overnight at 4 C. They were washed with PBS and then horseradish peroxidase-conjugated (H.R.P, sc-516102) mouse anti-rabbit IgG was used as secondary antibodies (sc-2357). After 2 hr incubation at room temperature, they were washed with PBS, they were soaked in ECL detection reagents (Amersham, UK) for 1 min. The signals of membranes were exposure to autoradiography film (Kodak, USA) and the results bands were quantified by ImageJ software (National Institutes of Health, USA) and normalized to the matching internal control.

### Ethical Considerations 

Ethical approval for this study was obtained from the Ethics Committee of Biomedical Research at Kharazmi University, Tehran, Iran (Code: IR.KHU.REC.1400.006). Following the approval from the Ethics Committee of Tabriz University of Medical Sciences, Tabriz, Iran permission was obtained to conduct the study at Al-Zahra hospital, Tabriz, Iran. Written informed consent was obtained from all participants.

### Statistical Analysis

GraphPad Prism 9.0 (GraphPad Software, San Diego, CA, USA) was used to create graphs and perform statistical analyses. One-way analysis of variance (ANOVA) was used for the effects of different doses of MGO at same time. Student's *t* test was used for differences between the control group and MGO-treated group. A p-value *<* 0.05 was considered statistically significant.

## 3. Results 

**Figure 1:**
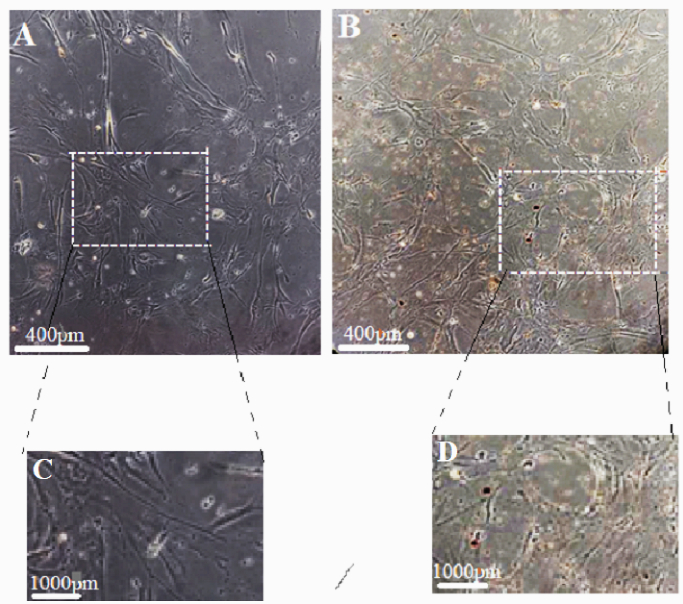
Magnetic graphene oxide (MGO) exposure changed morphologically and expanded the cell membrane processes of cumulus cells (CCs). On CCs surface treated with MGO, cells with expanded shapes, and filopodia and lamellipodia like extensions are observed. A and C, control groups, B and D, treated groups with MGO. A and B magnification: 100, C and D magnification: 400.

**Figure 2:**
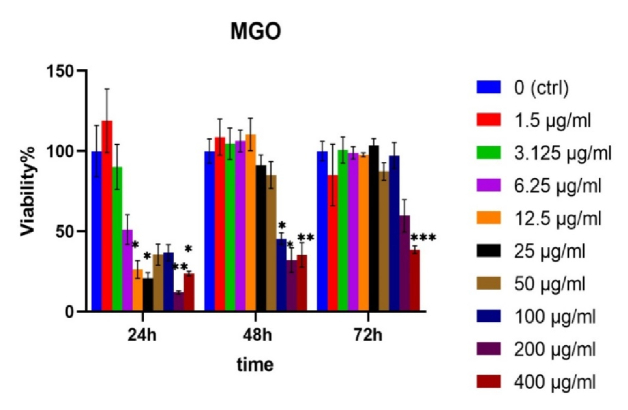
The effects of different concentrations of nano magnetic graphene oxide (MGO) on human CCs viability after 24, 48, and 72 hr. Repeated measures analysis of variance (ANOVA) was used to do statistical analysis. *P *<* 0.05, **P *<* 0.01, ***P *<* 0.001.

The results showed CCs treated with MGO have expanded shapes with higher processes like filopodia and lamellipodia extension compared to untreated group (Figure 1). This result is consistent with the results of MTT assay, which showed greater cell viability of CCs compared to the untreated surfaces. When exposed to MGO nanoparticles, not only did the survival rate of CCs decrease but also increased significantly in some concentrations after 24, 48, and 72 hr of incubation (Figure 2).

### Dose and time depended effect of MGO on cumulus cell viability

The findings of this study demonstrated that the effect of MGO on CCs viability was time and dose dependent. Findings of this study showed that after 24 hr of exposure to MGO at a concentration of 1.5 µg/ml, cell proliferation increased. However, with increasing concentrations, cell viability decreased, reaching 32.6% at 12.5 µg/ml (p < 0.05) and 9.11% at 200 µg/ml (p < 0.01). After 48 hr, MGO led to increased proliferation in CCs up to a concentration of 12.5 µg/ml. However, cell viability decreased thereafter, reaching 45.3% at 100 µg/ml (p < 0.05) and 32% at 200 µg/ml (p < 0.01). After 72 hr, cell viability in some concentrations, such as 3 and 25 µg/ml, was like the control (100%). However, it then decreased slightly, reaching 59.6% and 38.5% at 200 and 400 µg/ml, respectively (p < 0.001). According to the finding, the safe window for MGO activity is 48 hr (Figure 2).

### MGO reduced apoptosis in CCs

**Figure 3:**
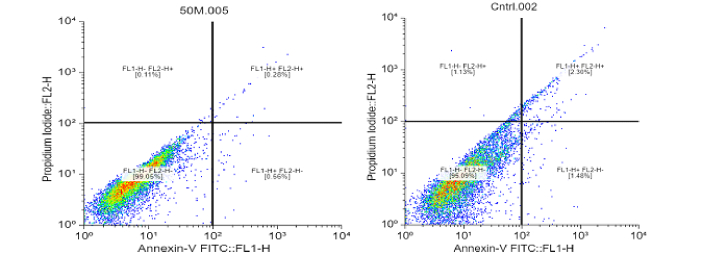
Flow cytometry quantitative analysis of cumulus cells after 48 hr treatment with MGO. The figure shows that magnetic graphene oxide treatment decreases the expression of apoptosis. In concentration of 50 µg/ml.

During apoptosis, phosphatidylserine is translocated from the inner layer to the outer layer of the cell membrane, and annexin binds to phosphatidylserine on the outer layer and is detected by flow cytometry. In addition, propidium iodide binds to fragmented DNA in the nuclei of dead cells and can also be detected by flow cytometry. Based on MTT assay findings, these 4 concentrations of MGO (3.125, 12.5, 50, and 100 µg/ml) were selected. The concentrations of 3.125, 12.5, and 50 µg/ml of MGO exhibited more than 87% cell viability. They were compared to a concentration of 100 µg/ml (near 50% viability) to identify the optimal dose that further reduces apoptosis. This optimal dose will be confidently used as the single dose for subsequent experiments (western blotting). As presented in table I, all concentrations of MGO (3.125, 12.5, 50, and 100 µg) elicited a reduction in apoptosis compared to the control group. Except for the 3.125 µg/ml concentration, apoptosis reduction at the remaining concentrations was statistically significant (p < 0.001). The percentage of necrosis cells was measured and the amount of necrosis at concentrations of 3.125, 12.5, 50, and 100 µg/ml were 0.33%, 0.30%, 0.11%, 0.13% compared with control group 1.13% (p < 0.0001). We found that nano-MGO treatment resulted in decreased apoptosis (Figure 3).

### Nrf2 protein level was upregulated by MGO in CCs

**Figure 4:**
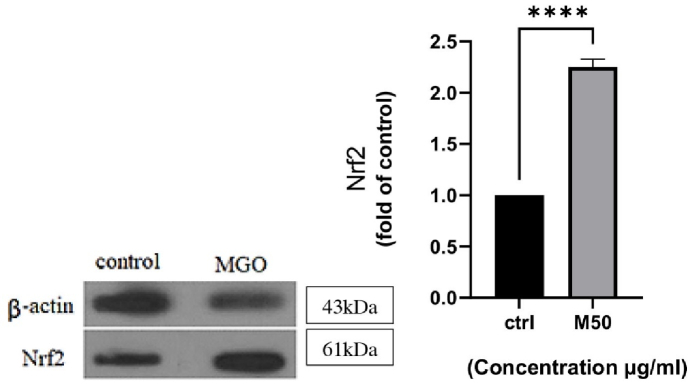
The expression of nuclear factor erythroid 2-related factor 2 (Nrf2) protein in cumulus cells exposed to 50 µg/ml concentration of MGO (right), representative blot (left). Student's *t* test was used to do statistical analysis. ****P *<* 0.001.

This panel showed the expression of Nrf2 proteins, components of the oxidative stress response pathway and antioxidant defense, which was increased after treatment with 50 µg/ml of MGO concentration. As seen in (Figure 4) this protein was upregulated by MGO more than 2-fold compared to the control (p < 0.0001), suggesting a protective and potentiation effects of MGO.

**Table 1:** Flow cytometry results measuring the levels of apoptosis and necrosis in CCs under the influence of MGO

**MGO concentration**	**3.125 µg/ml**	**12.5 µg/ml**	**50 µg/ml**	**100 µg/ml**	**Control**	**P-value**
**Viability (%)**	97.27 ± 0.24 (a)	98.29 ± 0.52	98.73 ± 0.32	98.81 ± 0.27	96.17 ± 1.08	< 0.01
**Apoptosis (%) (early + late)**	2.38 ± 0.22 (b)	1.54 ± 0.39	1.12 ± 0.28	1.04 ± 0.25	3.25 ± 0.52	< 0.001
**Necrosis (%)**	0.35 ± 0.02	0.34 ± 0.05	0.15 ± 0.04	0.15 ± 0.02	1.1 ± 0.10	< 0.0001
Data presented as Mean ± SD. One-way ANOVA test. Values (Mean ± S.D.) are from 3 independent experiments (n = 3). ANOVA (one way) was used to do statistical analysis. P-values indicates the level of significance between each concentration of MGO in the control group. (a), (b) There were no significant differences vs. control. CCs: Cumulus cells, MGO: Magnetic graphene oxide						

## 4. Discussion

The results showed CCs treated with MGO have expanded shapes with more processes such as filopodia and lamellipodia-like extensions, compared to the untreated group. Cell morphology is an important aspect of phenotype of a cell and is critical in the regulation of cell activities. This result was consistent with the results of MTT assay, which showed greater cell viability of CCs compared to the untreated surfaces. The expansion of cellular processes observed in this study indicates an increase in vital activities following an increase in survival rate. In many biological reactions, the interaction and communication of the cells with their environment are very important for their function. One of the ways to communicate is the presence of finger-like structures such as filopodia and lamellipodia. The results of the present study showed that MGO nanoparticles not only increased the viability of CCs at a concentration of 12.5 µg/ml, but it was not toxic even at concentrations of less than 100 µg/ml after 48, and 72-hr incubation, which indicated its excellent biocompatibility. The survival rates of the CCs in response to MGO at concentration of 12.5 µg/ml were different compared with control group for 48 hr. When the length of incubation time was extended from 48–72 hr, cell viability was reduced, with median inhibitory concentrations of 221, 438 and 392 mg/mL for MGO which indicates that CCs should not be exposed to MGO for more than 72 hr. As illustrated in figure 3 part 2, viability of CCs was gradually changed with the increased concentration of MGO (more than 50 µg/ml) and the extension of reaction time. In other words, concentrations of more than 50 µg/ml were toxic for CCs. Indeed, the potential biocompatibility and cytotoxicity of MGO are correlated with concentrations and incubation times. For example, it was shown that the adhesion capacity of bone marrow mesenchymal stem cells improved when they were incubated with 0.1 mg/ml MGO, while it was inhibited when the cells were exposed to 10 or 50 mg/ml MGO (7). Adhesion and proliferation of cells were gradually suppressed, and cellular morphology was significantly changed with the increased concentration of MGO and the extension of reaction time (16). The morphology cells revealed appreciable changes when the cells were treated with the dosage of 12.5 µg/ml MGO nanocomposites. Some studies showed that at high doses such as 100 µg/ml this nanoparticle is not toxic for dental pulp stem cells applications (13), while in mesenchymal stem cells derived from bone marrow, low doses such as 10 and 50 mg/mL were toxic (16). It could be concluded that cytotoxicity of some graphene oxides derivatives is dependent on the kinds of cells. The data of the present research, the time and concentration of MGO exposed to the CCs should be precise to increase the survival rate.

Graphene-based materials as a new class of antioxidants are shown to protect a variety of molecular targets from oxidation, and to be highly effective as hydroxyl-radical scavengers (10).

On the other hand, the use of assisted reproductive technology during IVFincreases the risk of free radical production (17, 18). Usually, free radicals are created during IVF, inducing gametes and embryo toxicity (10). One of which can be increasing the antioxidant activity of cells involved in IVF. Such free radicals may fight early oxidative stress by activating the nuclear factor erythroid Nrf2 signaling pathway. Our study provides novel insight into effect of MGO on CCs toxicity and an experimental rationale for the increase of female fertility through modulating the Nrf2 signaling pathway in vivo. The CCs play essential roles in protecting the oocytes from oxidant damage. Some reports have shown irregular patterns of antioxidant enzymes in the CCs of patients with polycystic ovary syndrome and endometriosis (19). Perhaps the increase of viability of CCs by MGO indicates its antioxidant role. Oocytes dose not synthesize enough levels of antioxidants and are dependent on the antioxidant secretion by CCs. However, in most cases of patients, the amount of total antioxidant in plasma decreases. In fact, the quality of the oocytes and embryo are related to the antioxidants effect of CCs. Based on the other results, GO derivatives do not have a negative effect on the survival rate of cells (20). Our results showed that MGO not only reduce the viability of CCs but also increase the survival rate of them at concentrations 50 µg/ml. The increase in the survival rate and the secretion of antioxidants Nrf2 protein from CCs after treatment with MGO is very important because they support oocytes and provide essential antioxidants such as Nrf2 for oocytes. It seems one of the reasons for the increase in the viability of CCs induced by MGO is related to the increase in the expression of Nrf2 protein. The increase in the expression of Nrf2 is a sign of promoting cyto-protective activity of CCs induced by MGO (21). Based on these results, we speculated that MGO plays an antioxidant and protective role in increasing the viability of CCs through Nrf2 pathway.

## 5. Conclusion

In Conclusion, MGO nanoparticles may have promising applications as carriers for drug or molecule delivery targeting CCs. We showed that the MGO nanoparticles are nontoxic and biocompatible and has antioxidant property in vitro, with biomedicine application.

##  Data Availability

Data supporting the findings of this study are available upon reasonable request from the corresponding author.

##  Author Contributions

F. Kabiri: Data curation, writing-review, and editing. T. Foroutan: Conceptualization, investigation, methodology, project administration, supervision, visualization, writing-original draft, writing-review, and editing. M. Pashaiasl: Data curation and investigation.

##  Conflict of Interest

The authors declare that there is no conflict of interest.
