# Correction to “Magnetic graphene oxide increases the biocompatibility and nuclear factor erythroid 2-related factor 2 antioxidant of human cumulus cells: A lab-trial study” [Int J Reprod BioMed 2024; 22: 709–716]

**DOI:** 10.18502/ijrm.v23i3.18769

**Published:** 2025-06-10

**Authors:** Fahimeh Kabiri, Tahereh Foroutan, Maryam Pashaiasl

The publisher has been informed of an error that occurred on pages 709 (Vol. 22; No. 9) which the p-value sign mistakenly given as > instead of < . The publisher wishes to apologize for this error. The online version of the article has been updated on March 31, 2025 and can be found at https://doi.org/10.18502/ijrm.v22i9.17475.

